# X-ray, digital tomographic fusion, CT, and MRI in early ischemic necrosis of the femoral head

**DOI:** 10.1097/MD.0000000000036281

**Published:** 2024-01-12

**Authors:** Hong Ge, Zhuhai Wang, Jiangang Zhang

**Affiliations:** a Radiology Department, The Third Hospital of Hebei Medical University, Qiaoxi District, Shijiazhuang, China.

## Abstract

To investigate the imaging performance of radiography, digital tomographic fusion (DTS), computed tomography (CT), and magnetic resonance imaging (MRI) in the diagnosis of early avascular necrosis of the femoral head (ANFH). A total of 220 patients with ANFH who visited our hospital from January 2020 to January 2022 were included in the study. X-ray, DTS, CT, and MRI examinations of both hips were performed for all patients. The trabecular structure, bone density changes, femoral head morphology, and joint space changes were observed using the aforementioned imaging modalities. The staging was performed according to the Association Research Circulation Osseous (ARCO) criteria. The diagnostic detection rate of each imaging modality, and the sensitivity, specificity, positive predictive value, and negative predictive value of each examination for diagnosing early ANFH were calculated and compared. Patients were diagnosed with stage I (n = 65), stage II (n = 85), stage III (n = 32), and stage IV (n = 38) ANFH. For MRI, the detection rate (97.7%), sensitivity (94.7%), specificity (88.6%), positive predictive value (95.9%), and negative predictive value (92.5%), for diagnosing early ANFH, were significantly higher than those of other imaging methods (*P* < .05). MRI is the most accurate and sensitive imaging method for diagnosing early ANFH and has important clinical applications.

## 1. Introduction

Avascular necrosis of the femoral head (ANFH) is a common orthopedic disease characterized by bone cell apoptosis due to impaired blood supply to the femoral head, structural changes in the bone trabeculae, bone collapse, femoral head deformation, and ultimately irreversible changes, such as bone destruction and joint space narrowing, occur.^[1,2]^ Epidemiological studies have shown that in the United States, there are over 25 000 new cases of ANFH annually, accounting for 14.9% of the approximately 480 000 total hip replacement surgeries performed each year.^[3]^ Early ANFH symptoms are usually not obvious, and some patients have mild hip pain, followed by progression to joint mobility disorders, walking difficulties, secondary osteoarthritis, and eventually even joint replacement surgery, which places a heavy burden on patients and families.^[4]^ Therefore, early diagnosis of ANFH and timely intervention are particularly important for maintaining joint function, improving the quality of life, and enhancing the prognosis of the patient.^[5]^

Currently, the diagnosis of ANFH in clinical practice relies on noninvasive imaging methods, such as X-ray, computed tomography (CT), and magnetic resonance imaging (MRI).^[6,7]^ Digital tomography fusion (DTS) is used to obtain a clear image of the target area by blurring the image outside the observation area after a one-time scan using an X-ray bulb, and the image is reconstructed using computer post-processing software.^[8,9]^ We analyzed and compared the X-ray, CT, MRI, and DTS imaging performances in diagnosing ANFH at different stages, to evaluate the value of various imaging modalities for ANFH staging and early ANFH.

## 2. Methods

### 2.1. General information

A total of 220 patients (men: 125; women: 95; mean age: 43.8 ± 11.7 [range: 21–60] years) with ischemic necrosis of the femoral head who visited our hospital from January 2020 to January 2022 were included in this study. The mean duration of disease was 6.9 ± 2.3 (range: 1–38) months. The lesions were located in the left hip in 103 patients and the right hip in 117 patients. There were 52 cases of definite hip trauma, 60 cases of long-term alcohol abuse, 50 cases of long-term glucocorticoid use, and 58 patients with unknown causes of onset. We explained the research content and process in detail to all patients and their families. All patients volunteered to participate in this study and provided written informed consent. This study was approved by the Ethics Committee of the Third Hospital of Hebei Medical University (Approval Number: 2016126).

### 2.2. Inclusion and exclusion criteria

The inclusion criteria were as follows: unilateral onset; clinical manifestations, such as hip pain and discomfort, lower limb mobility disorder, and positive 4-character sign on examination; availability of complete clinical data; and willingness to cooperate for follow-up.

The exclusion criteria were as follows: previous hip surgery; serious comorbid conditions with contraindications to imaging; psychiatric or psychological diseases with cognitive impairment and inability to cooperate with imaging; pregnancy or lactation; and malignant and advanced tumors.

### 2.3. Imaging

X-ray, DTS, CT, and MRI examinations of both hips were performed on all patients. The procedures were as follows: X-ray examination: a double-plate DR camera system (Philips) was used to obtain orthopantomograms of the hip joint. CT examination: A Siemens Sensation 64-row spiral CT scanner (Siemens Medical Solutions, Forchheim, Germany) with parameters set at 120 kV, 220 mAs, a layer thickness of 1 mm, and layer spacing of 5 mm were used; the patient was placed in the supine position and scanned continuously from the upper acetabulum to the lower femoral head. MRI examination: Siemens Magnetom 3.0T superconducting MRI scanner with conventional sagittal spin-echo T1-weighted (WI; TR/TE: 500 ms/25 ms) and T2-WI (TR/TE: 3500 ms/90 ms) image was used. The patient was placed in the supine position, and the femoral head was scanned continuously. DTS examination: The Japanese Shimadzu large-plate multifunctional digital fluoroscopy system (Sonialvision Safire 17, Shimadzu, Japan) was used, with scanning parameters set to 85 kV and 400 mA. The femoral head was scanned and transmitted to the workstation for image reconstruction; the height of the central level of the post-processing workstation reconstruction was 120 mm, with a layer thickness of 0.5 mm.

### 2.4. Clinical staging

Staging was performed according to the ANFH staging criteria developed by the Association Research Circulation Osseous (ARCO). There are 5 stages: Stage 0: pathological findings show femoral head necrosis with negative imaging findings. Stage I: MRI shows abnormalities but no abnormalities on radiography. Stage II: abnormal X-ray but no collapse, with abnormal MRI findings. Stage III: crescentic sign, femoral head collapse, or subchondral fracture manifestation. Stage IV: significant collapse of the femoral head and narrowing of the joint space. According to these staging criteria, stages I and II are considered early; whereas, stage III is the middle stage and stage IV is the late stage. Two senior diagnostic imaging physicians staged the lesions by evaluating the imaging data of the various modalities. In cases of difference in opinions, a discussion was held until a consensus was achieved. The diagnostic criteria of MRI are: stage I, double line sign and high T2 weighted signal; stage II, increased T1 and T2 weighted signals; stage III decreased T1 weighted signals and increased T2 weighted signals; and stage IV, local collapse and deformation of the lesion and decreased T1 and T2 weighted signals.

### 2.5. Image summary and data analysis

All acquired images were pooled and analyzed to observe the bone trabecular structure, bone density changes, femoral head morphology, joint gap changes, and other indicators. The diagnostic detection rate of each imaging modality as well as the sensitivity, specificity, positive predictive value, and negative predictive value for the diagnosis of early ANFH were calculated and compared.

### 2.6. Statistical analysis

Statistical analysis was performed using Statistical Package for the Social Sciences (SPSS) version 21.0 (IBM SPSS Statistics for Windows, Version 21.0., Armonk, NY, IBM Corp. Count data are expressed as the number of cases (n) and percentages (%), and measurement data are expressed as means ± standard deviations (x ± s). The count data were compared using the χ^2^ test or Fisher exact test. The *t*-test was used for normally distributed data; whereas, the signed rank-sum test was used for measurement data not conforming to normal distribution. The test level used was α = 0.05, and statistical significance was set at *P* < .05.

## 3. Results

### 3.1. Comparison of imaging performance and staging

A total of 139 cases were diagnosed using X-rays (detection rate: 63.2%). Thirty-three patients at stage I exhibited blurred bone trabeculae, 33 cases at stage II revealed limited osteoporosis and bony cystic lesions without femoral head collapse, 25 cases at stage III displayed local sclerosis with crescentic signs and femoral head collapse but normal joint space, and 29 cases at stage IV exhibited deformed and collapsed femoral head and narrow joint space.

A total of 157 patients were diagnosed using CT (detection rate: 71.4%). Thirty-nine cases at stage I showed blurred bone trabeculae, heterogeneous density, and textural disorder; 53 cases at stage II exhibited limited bone cystic changes and sclerotic areas of varying sizes; 29 cases at stage III possessed subchondral fractures and crescent formation; and 36 cases at stage IV showed femoral head collapse with visible free bodies and associated degenerative osteoarthritis.

A total of 215 patients were diagnosed using MRI (detection rate: 97.7%). Sixty-four cases at stage I showed T1-WI linear low signal and T2-WI high signal; 84 cases at stage II exhibited T1-WI heterogeneous low signal, T2-WI moderate slightly high signal, surrounded by heterogeneous low signal, and a bilinear sign; 32 cases at stage III displayed T1-WI banded low signal, T2-WI moderate or high signal, joint capsule hypertrophy, and joint cavity effusion; and 35 cases at stage IV exhibited articular cartilage destruction and acetabular sclerosis on T1-WI and T2-WI.

A total of 168 cases were diagnosed with DTS (detection rate: 76.4%). Of these, 52 cases at stage I showed disorganized and blurred bone trabeculae and reduced local bone density; 73 cases at stage II exhibited speckled hypointense shadows in the weight-bearing area of the femoral head and disorganized bone trabeculae; 20 cases at stage III displayed scattered high-density shadows in the femoral head and collapse of the joint surface, which was seen as a crescentic sign; and 23 cases at stage IV exhibited loss of femoral head morphology and joint space narrowing with degenerative osteoarthritis.

The detection rate achieved by MRI was significantly higher than those of the other imaging modalities (*P* < .05), and the diagnostic results are summarized in Table 1.

**Table 1:** Diagnostic results of X-ray, CT, MRI, and DTS.

Imaging	Early		Mid-term	Late-stage	Total	Detection rate
	Stage I	Stage II	Stage III	Stage IV		
X-ray	33	52	25	29	139	63.2%
CT	39	53	29	36	157	71.4%
MRI	64	84	32	35	215	97.7%
DTS	52	73	20	23	168	76.4%

CT = computed tomography, DTS = digital tomographic fusion, MRI = magnetic resonance imaging.

### 3.2. Comparison of the diagnostic efficacy in early ANFH

According to ARCO staging, 220 patients were diagnosed with stage I (n = 65); stage II (n = 85); stage III (n = 32); and stage IV (n = 38) ANFH. There were 150 cases of early ANFH (stages I and II) and 70 cases of mid- and late-stage ANFH (stages III and IV).

The efficacy of each imaging modality in diagnosing early ANFH is summarized in Table 2. CT exhibited 38.7% sensitivity (58/150), 35.7% specificity (25/70), 63% positive predictive value (58/92), and 38.5% negative predictive value (25/65). MRI achieved 94.7% (142/150) sensitivity, with 88.6% specificity (62/70), 95.9% positive predictive value (142/148), and 92.5% negative predictive value (62/67). The sensitivity of DTS diagnosis was 78.7% (118/150), with 50% specificity (35/70), 94.4% positive predictive value (118/125), and 81.4% negative predictive value (35/43). The sensitivity and specificity of MRI for the diagnosis of early ANFH were significantly higher than those of the other imaging modalities (*P* < .05).

**Table 2:** Diagnostic efficacy of X-ray, CT, MRI, and DTS in early ANFH.

ARCO Staging	X-ray		CT		MRI		DTS	
	(+)	(−)	(+)	(−)	(+)	(−)	(+)	(−)
(+)	40	31	58	40	142	5	118	8
(−)	45	23	34	25	6	62	7	35
Total	85	54	92	65	148	67	125	43

ARCO stages I and II are considered early lesions (+); ARCO stages III and IV are considered non-early lesions (−).

ANFH = avascular necrosis of femoral head, ARCO = Association Research Circulation Osseous, CT = computed tomography, DTS = digital tomographic fusion, MRI = magnetic resonance imaging.

## 4. Discussion

The etiology of ANFH is divided into traumatic and non-traumatic factors. Traumatic etiologies are mainly due to sequelae arising from femoral neck fractures, whereas non-traumatic etiologies include excessive alcohol intake, steroid medications, autoimmune diseases, and certain blood disorders such as hypofibrinolysis.^[10]^ Both alcohol- and steroid-induced ANFH are dose-dependent; however, the underlying mechanism is unclear and may be related to impaired phospholipid metabolism, vasculitis, and microthrombosis.^[11,12]^ Among the 220 patients included in this study, the 3 main causes of morbidity were long-term alcohol abuse (60 [27.3%]), hip trauma (52 [23.6%]), and long-term use of glucocorticoids (50 [22.7%]), which are consistent with previous reports.

Radiography is still considered essential for the diagnosis of ANFH and usually requires ortho- and lateral views, which are easy to perform and inexpensive.^[13]^ From early to late disease, the typical features of X-rays are sclerosis of the femoral head, “crescentic sign” consistent with subchondral fracture, the collapse of the femoral cortex, narrowing of the joint space, osteoarthritis, and joint dislocation^[14]^ presented as atypical imaging features, even without trabecular sparing or cystic changes. In this study, the detection rate of X-rays was 63.2%, and the sensitivity for diagnosing early ANFH was only 26.7%. Although it is not sensitive to the early-stage changes, late manifestations are obvious, and additional imaging evaluations can be avoided. CT examination possesses a high spatial resolution and is more sensitive than X-rays in detecting typical manifestations of osteonecrosis. On CT, ANFH manifests from the early to advanced stages of the disease as bone trabecular sparing, femoral head sclerosis, subchondral fracture, bone collapse, and joint deformity, as the disease progresses. Previously, researchers compared the staging performance of CT and MRI in ANFH and reported that CT was more effective in showing the bone structure, whereas MRI was more effective in displaying abnormal changes in the bone marrow.^[15]^ In the present study, the sensitivity of CT in diagnosing early ANFH was 38.7%; therefore, CT examination was more often used for the diagnosis of intermediate and advanced lesions and assessment of the area of osteonecrosis.

Many scholars regard MRI as the gold standard imaging examination for early ANFH, and in this study, we confirmed that MRI had a detection rate of 97.7% and the sensitivity of diagnosing early ANFH was as high as 94.7%.^[16]^ A previous study in animal models revealed that bone marrow signal changes within 1 week after trophoblastic vessel injury of the femoral head can be detected as abnormalities on MRI.^[17]^ The typical MRI presentation from the early to late stages is a T1-WI showing the area of osteonecrosis surrounded by a “band” of low-intensity areas corresponding to a reactive sclerotic rim around the diseased bone marrow. Subchondral fractures appear as smooth low-signal intensity lines on T1-WI depressed toward the articular surface, which may appear crescentic, serpentine, annular, or wedge-shaped depending on the extent of necrosis. The DTS technique reconstructs the image using computer software, which clearly shows the target area by blurring the surrounding tissues, enabling the observation of subtle lesions of anatomical structures without the influence of overlapping surrounding tissues. Therefore, the spatial resolution of DTS is higher than that of CT, whereas the ionizing radiation dose is much lower. DTS reveals changes in bone trabeculae, bone density, bone cortical continuity, femoral head morphology, and joint space; however, its detection rate (76.4%) is higher than that of CT examination, and the cost is relatively low.

Pathophysiologically, ANFH can be divided into ischemic and regenerative phases.^[17]^ The onset of ischemia is difficult to determine because it predates positive imaging and clinical manifestations; it remains unclear whether the origin of ischemia is in the vasculature of the trochoid epiphysis or the perichondral stromal vessels. At this stage, the bone structure remains unchanged and may appear normal on X-ray and CT, whereas early bone marrow disturbances may be detected on MRI.^[18]^ Subsequently, bone marrow mesenchymal stem cells proliferate and differentiate into osteoblasts, resulting in new homogeneous bone, and osteoclasts undergo bone resorption, indicating the beginning of the regenerative phase. During this phase, osteoclasts begin to crawl for replacement action, which may appear as a subchondral fracture on an X-ray. Both new bone production and dead bone resorption require the transport of nutrients through blood vessels; therefore, new blood vessels gradually penetrate to occupy the subchondral bone, and collapse occurs when the weight-bearing area of the femoral head is replaced by fibrous tissue, followed by the formation of intrachondral bone scabs and osteoarthritic changes. We demonstrated that MRI had the highest sensitivity and specificity for diagnosing early ANFH, at 94.7% and 88.6%, respectively, which also validates the pathological process of ANFH. In addition, despite the slightly higher cost, MRI examinations are free of ionizing radiation, relatively safer, and possess advantages over X-rays, CT, and DTS.

## 5. Conclusion

In conclusion, MRI exhibited the highest detection rate for ANFH; the sensitivity and specificity of diagnosing early ANFH were also significantly higher than those of the other imaging modalities; the examination process is safe, making it ideal for detection of early ischemic necrosis of the femoral head. Therefore, MRI has important applications with considerable significance for guiding treatment and improving the prognosis of patients.

## Acknowledgments

We would like to thank Editage (www.editage.cn) for English language editing.

## Author contributions

**Conceptualization:** Hong Ge.

**Data curation:** Zhuhai Wang.

**Formal analysis:** Jiangang Zhang.
